# Capturing sleep–wake cycles by using day-to-day smartphone touchscreen interactions

**DOI:** 10.1038/s41746-019-0147-4

**Published:** 2019-07-29

**Authors:** Jay N. Borger, Reto Huber, Arko Ghosh

**Affiliations:** 1 Institute of Psychology, Cognitive Psychology Unit, Leiden University, Leiden, The Netherlands; 2 Child Development Center, University Children’s Hospital Zurich, Switzerland & Department of Child and Adolescent Psychiatry and Psychotherapy, Psychiatric Hospital University of Zurich, Zürich, Switzerland

**Keywords:** Human behaviour, Biomarkers, Risk factors

## Abstract

Body movements drop with sleep, and this behavioural signature is widely exploited to infer sleep duration. However, a reduction in body movements may also occur in periods of intense cognitive activity, and the ubiquitous use of smartphones may capture these wakeful periods otherwise hidden in the standard measures of sleep. Here, we continuously captured the gross body movements using standard wrist-worn accelerometers to quantify sleep (actigraphy) and logged the timing of the day-to-day touchscreen events (‘tappigraphy’). Using these measures, we addressed how the gross body movements overlap with the cognitively engaging digital behaviour (from *n* = 79 individuals, accumulating ~1400 nights). We find that smartphone use was distributed across a broad spectrum of physical activity levels, but consistently peaked at rest. We estimated the putative sleep onset and wake-up times from the actigraphy data to find that these times were well correlated to the estimates from tappigraphy (R^2^ = 0.9 for sleep-onset time and wake-up time). However, actigraphy overestimated sleep as virtually all of the users used their phones during the putative sleep period. Interestingly, the probability of touches remained greater than zero for ~2 h after the putative sleep onset, and ~2 h before the putative wake-up time. Our findings suggest that touchscreen interactions are widely integrated into modern sleeping habits—surrounding both sleep onset and waking-up periods—yielding a new approach to measuring sleep. Smartphone interactions can be leveraged to update the behavioural signatures of sleep with these peculiarities of modern digital behaviour.

## Introduction

There is a well-recognised need for tracking sleep in patients, as well as in the general population. This rush to quantify sleep is partly driven by the increased awareness that sleep is crucial for cognitive performance and well-being. Body movements offer an easy proxy for sleep measurements. Essentially, users wear an accelerometer at the wrist or ankle and the recorded accelerations i.e., body movements are automatically converted into estimates of sleep.^1,2^ This popular method relies on the sharp drop in motility at sleep onset and the sharp rise with wakefulness.^1,3^ The body movements recorded at the joints may involve different levels of cognitive engagement—from reflexes to the postural control accompanying fine motor control. However, the extent of cognitive engagement does not enjoy a simple linear relationship with the movements recorded at the joints. For instance, the now common fine finger movements on the touchscreen are cognitively engaging, but they may result in no or negligible signal deflections at the wrist. This lack of a simple relationship and the widespread use of smartphones in modern behaviour warrant an up-to-date perspective on tracking sleep based on motor activity.

In general, modern digital interactions offer unprecedented opportunities to quantify behaviour in the real world with major repercussions for sleep. For instance, the timing of social messaging such as on Twitter can be used to elaborate the diurnal behavioural patterns.^4,5^ This measure of online activity is limited in terms of capturing the behaviour continuously. For instance, only a fraction of the digital behaviour occurs via the social messaging server. Another approach has focused on the mobile device itself, and sleep–wake cycles can be inferred by machine-learning algorithms that use the hardware state of the smartphone (i.e., phone on the charger and the screen being on or off) as inputs and sleep diaries as the ground truth.^6^ This ‘black-box’ approach is not designed to improve the fundamental understanding of motor behaviour and sleep, but it does underscore the value of capturing data from the smartphone sensors in the context of sleep. However, there is a large gap between the accuracy of phone-based sleep detection and the objective measures of sleep.^7^ Regardless of the current limitations and pending validations of these novel approaches, they do promise an economical, easy to administer and highly scalable measure of sleep based on existing sensors in contrast to approaches that require extra sensors as used in standard actigraphy.

In this study, we used standard wrist-worn actigraphy to quantify sleep–wake cycles and in parallel recorded the timestamps of smartphone touchscreen interactions. Although smartphones have built-in accelerometers capable of monitoring body movements—as long as it is carried by the user—the wrist-worn approach ensures that all of the movements are independently and continuously recorded, including when at asleep. We focused on the Cole–Kripke algorithm, which is well studied and widely used to infer sleep from the body movements. This algorithm has been validated against the gold standard or direct measure of sleep using polysomnography.^3,8–10^ By merging these distinct measures, we quantify the patterns of overlap between smartphone behaviour and sleep, and validate a novel approach to measure sleep derived from the smartphone interactions alone.

## Results

### Physical activity and smartphone usage

**Fig. 1:**
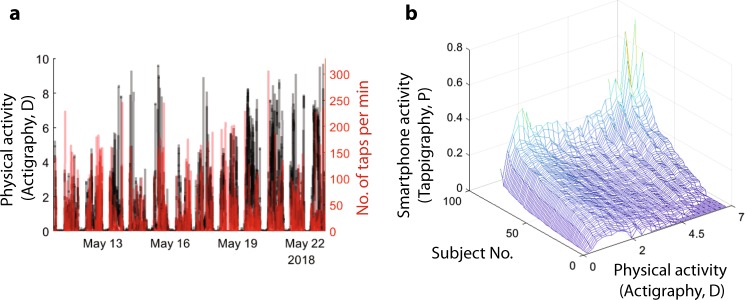
The relationship between smartphone touchscreen interactions and gross movements. **a** The data from a single participant showing the extent of the overlap between smartphone interactions quantified using an App running in the background (‘tappigraphy’) and overall physical activity measured at the wrist and quantified using an actigraphy algorithm, where *D* ≤ 1 is indicative of physical rest. **b** The probability of smartphone interactions in 1-minute bins at different levels of physical activity quantified in steps of 0.25 D. The subjects are sorted according to the amount of smartphone activity at *D* = [0–0.25]

We estimated the variations in smartphone interactions at the different levels of physical activity (Fig. 1). In keeping with our goal of better understanding sleep–wake cycles, we quantified the activity in terms of the actigraphy ‘*D*’ values, where *D* is proportional to the sum of the acceleration at any given minute and the surrounding minutes. Importantly, *D* < 1 corresponds to sleep in the Cole–Kripke algorithm, whereas *D* > 7 corresponds to high actigraphy activity which could result from a range activities from a brisk walk to biking, and from riding a bus on a bumpy road to climbing stairs.^3^ In all of the participants, the probability of observing smartphone interactions increased when at physical rest (*D* ≈ 1, Fig. 1). A subset of participants showed a bimodal distribution such that the smartphone interactions frequently occurred both at low and high actigraphy values. In 42/79 participants, the smartphone interactions were maximum between the *D* values of 0 and 2, and 34/79 participants show the maximum values at greater than 6. Interestingly, the smartphone behaviour at physical rest was related to the behaviour with high actigraphy value, such that the higher the smartphone usage at complete physical rest (0.25 ≥ *D* ≥ 0) the higher the phone usage when high actigraphy activity (*D* > 7, *R*^2^ = 0.14, *β* = 1.13, *t*(72) = 3.33, *p* = 0.001). For subjects eliminated from the analysis, please see Supplementary Table.

### Comparison of tappigraphy-based sleep–wake estimations to standard actigraphy and sleep diaries

**Fig. 2:**
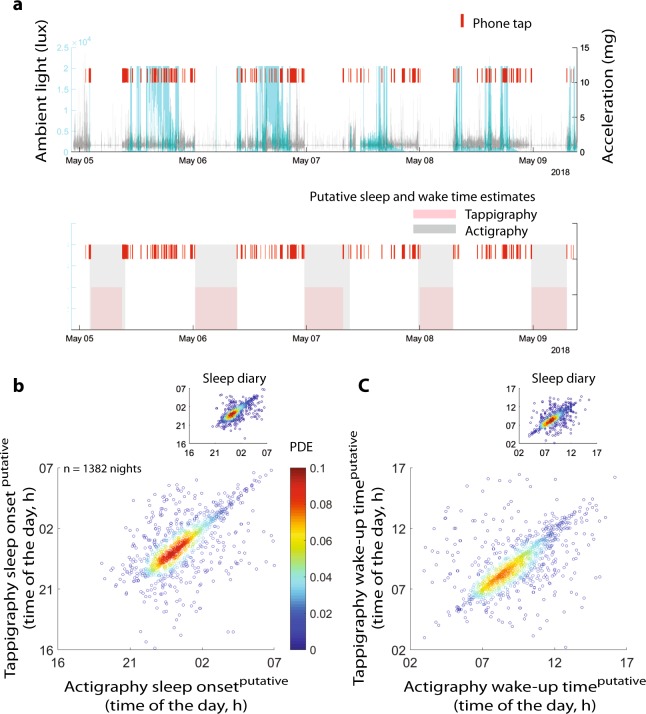
Comparison of tappigraphy-based sleep estimates with that of actigraphy. **a** Actigraphy watches were used to quantify the amount of ambient light, near-body temperature (not shown) and the body movements. The smartphone touches were simultaneously recorded by using an App running in the background. We used the Cole–Krpike algorithm to extract the putative sleep times from actigraphy, and a new algorithm was designed to extract the sleep times from the smartphone touches (‘tappigraphy’). Reflective example of putative sleep and wake estimates is presented in ‘**a**’ bottom panel. The relationship between the putative sleep-onset times (**b**) and wake-up times (**c**) determined by using actigraphy versus tappigraphy. The data were pooled by concatenation across all the subjects. The time of the day is in local time, and the inserts show the relationship between tappigraphy versus sleep diaries. PDE probability density estimate based on kernel smoothing function

The high probability of smartphone touches at physical rest raised the opportunity that the putative sleep times can be simply estimated by observing the gaps in smartphone usage. We compared two standard methods used to estimate sleep with that of a new tappigraphy-based algorithm based on the gaps in smartphone usage. Pooling all of the measurements together (by concatenating the sleep-related estimates obtained from each participant), we found strong correlations between the putative sleep times determined by actigraphy versus tappigraphy (Fig. 2). For the sleep-onset times, a linear fit with a slope ≈1 well captured the relationship (*R*^2^ = 0.84, *β* = 0.93, *t*(1380) = 85.2, *p* < 0.0001). For the wake-up times, there was a strong correlation between the actigraphy (*x*) versus tappigraphy (*y*) estimates as well (*R*^2^ = 0.90, *β* = 0.90, *t*(1380) = 111.4, *p* < 0.0001). In terms of sleep duration (for non-concatenated *p-*values from each subject see Supplementary Fig. 1), tappigraphy consistently underestimated sleep given that the slope was substantially below 1 (*R*^2^ = 0.28, *β* = 0.49, *t*(1322) = 22.7, *p* = 1 × 10^−98^). This regression yielded an intercept of 3.9 (*p* < 0.0001) and *x* = *y* was at 7.6 h, suggesting that the tappigraphy sleep durations <7.6 h are underestimates of sleep. This could not be simply explained by the fact that the data used were from the left wrist, whereas the phones may be handled by the right. Using the right wrist movements, we again found a biased estimation of sleep duration (*R*^2^ = 0.30, *β* = 0.51, *t*(1318) = 23.8, *p* = 1.5 × 10^−104^ with an intercept of 3.8, *p* < 0.0001). A similar pattern was found when comparing tappigraphy to sleep diaries. For sleep-onset times and wake-up times, a linear fit with a slope ≈1 well captured the relationship between the two approaches (for sleep-onset times: *R*^2^ = 0.89, *β* = 0.99, *t*(1034) = 92.9, *p* < 0.0001 and for wake-up times: *R*^2^ = 0.94, *β* = 0.99, *t*(1108) = 136.0, *p* < 0.0001). As in actigraphy, the sleep duration was underestimated by tappigraphy (*y*) versus diary (*x*, *R*^2^ = 0.59, *β* = 0.88, *t*(1023) = 38.5, *p* = 4.71 × 10^−201^), and the regression model had an intercept of 1.39 (*p* = 2.36 × 10^−14^). It is interesting to note that how the sleep diary (*y*) related to actigraphy (*x*). The regression model was captured with a slope ≈0.5, suggesting subjects reported shorter durations compared with what was determined by actigraphy (*R*^2^ = 0.36, *β* = 0.47, *t*(1091) = 24.6, *p* = 7.42 × 10^−107^). Finally, we addressed whether the sleep diary on the smartphone may have consistently occurred right before bedtime or right after rise time (1 entry was made per day) to contaminate the tappigraphy algorithms, but no specific reporting pattern was observed, and the reports were scattered through all times of the day (Supplementary Fig. 2).

### Inter-individual differences in actigraphy and tappigraphy-based sleep–wake estimations

**Fig. 3:**
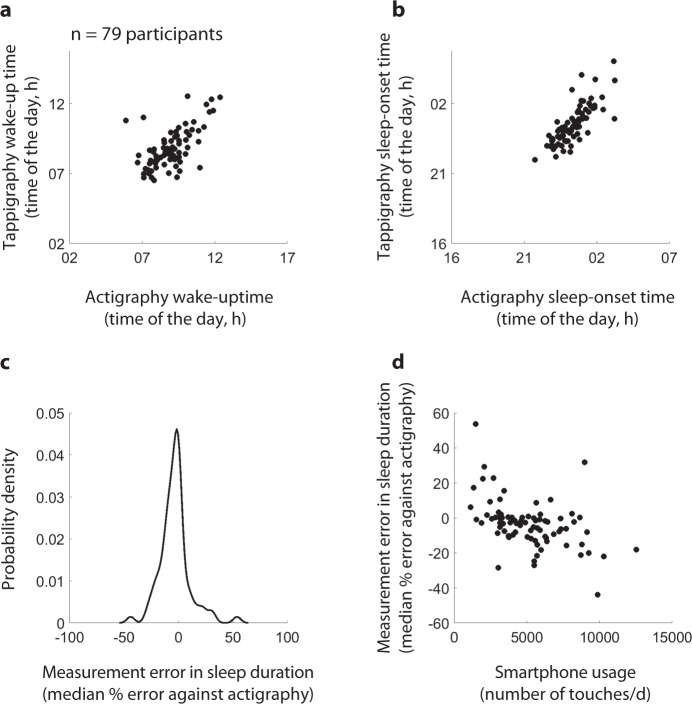
Inter-individual differences in tappigraphy and actigraphy sleep estimates. The individual central tendencies (median) of actigraphy-based putative wake-up (**a**) and sleep (**b**) times were well correlated to the putative times determined using tappigraphy. **c** The distribution of median measurement error (estimated from each individual) in tappigraphy considering actigraphy as ground truth observed in the sampled population. **d** The relationship between the individualised measurement error and smartphone usage in the sampled population

A key question is how engaged must any user be on the smartphone for the tappigraphy-based metrics to accurately reflect sleep. Considering the actigraphy-based measures as ground truth, we found that the median sleep-onset times and wake-up times derived from tappigraphy were well correlated to the values obtained from actigraphy (Fig. 3, for sleep onset: *R*^2^ = 0.72, *β* = 0.97, *t*(77) = 14.1, *p* = 4.45 × 10^−23^ and for wake-up times: *R*^2^ = 0.60, *β* = 0.83, *t*(77) = 10.7, *p* = 6.00 × 10^−17^). Next, we determined the median percentage error in estimating sleep duration against the actigraphy values to find the median absolute error to be 7.2%, and median error at −2.8%, i.e., in the majority of the sampled population sleep was underestimated (negative error) by tappigraphy. Note that these negative errors may well mean that actigraphy overestimates true sleep durations rather than tappigraphy underestimates the duration. This was further confirmed when comparing the population means derived by using actigraphy (8.5 h ± 0.94 STD) vs. tappigraphy (8.1 h ± 1.1 STD, *t*(78) = 2.4, *p* = 0.02). Interestingly, the errors were strongly related to smartphone usage—such that tappigraphy overestimated sleep in comparison with actigraphy for users who generated a low number of touchscreen touches per day, and the errors were reversed for the high smartphone users (*R*^2^ = 0.27, *β* = −0.0023, *t*(77) = −5.23, *p* = 1.4 × 10^−6^). With the 0-error intercept set at ≈3200 touches per day. This value offers a guideline on the extent of engagement needed to estimate sleep using tappigraphy. As the number of available days of measurement varied from 5 to 31 days per individual, we opportunistically addressed whether the errors were linked to the number of days of measurement, and this was not found to be the case (*R*^2^ = 0.02, *β* = −0.12, *t*(77) = −0.62, *p* = 0.53).

As sleep may vary from one night to the next, we measured the intra-individual variation in sleep duration (CoV) to find that the actigraphy versus tappigraphy values were correlated (*R*^2^ = 0.35, *β* = 0.57, *t*(77) = 6.13, *p* = 3.6 × 10^−8^). Finally, we exploited the demographic information to address whether the inter-individual differences in sleep durations and sleep CoV could be explained by the amount of phone usage (measured as number of touches per day), age, gender (dummy variable), height and weight. Age, height and weight were normally distributed, but the sample was focused on a rather narrow age range (mean age was 23 ± 2.6 STD). For the actigraphy-based sleep duration, the overall multiple regression model was not significant (*R*^2^ = 0.03, *F*(5, 71) = 0.44, *p* = 0.82). When using sleep CoV as the dependent variable, the full regression model was significant (*R*^2^ = 0.237, *F*(5, 71) = 3.38, *p* = 0.001), and the variation reduced with age (*β* = −0.01, *t*(71) = −2.08, *p* = 0.04, *p* > *α* post Bonferroni correction) and increased with weight (*β* = 0.003, *t*(71) = 3.02, *p* = 0.004). Next, we performed the same analysis using the sleep duration and sleep CoV values derived from tappigraphy. For the sleep duration, the overall model was highly significant (*R*^2^ = 0.30, *F*(5, 71) = 6.17, *p* = 8.39 × 10^−5^). The higher the phone usage the shorter the duration (*β* = −0.0002, *t*(71) = −4.71, *p* = 1.2 × 10^−5^), and the larger the weight the shorter the duration (*β* = −0.023, *t*(71) = −2.09, *p* = 0.04, *p* > *α* post Bonferroni correction). For sleep CoV, the overall model was significant (*R*^2^ = 0.23, *F*(5, 71) = 4.25, *p* = 0.002). The higher the phone use the lower the variation (*β* = −1.7 × 10^−5^, *t*(71) = −3.19, *p* = 0.002), and females were less variable than males (female = 1, *β* = −0.09, *t*(71) = −3.03, *p* = 0.003).

### Smartphone usage in actigraphy-derived ‘sleep’

**Fig. 4:**
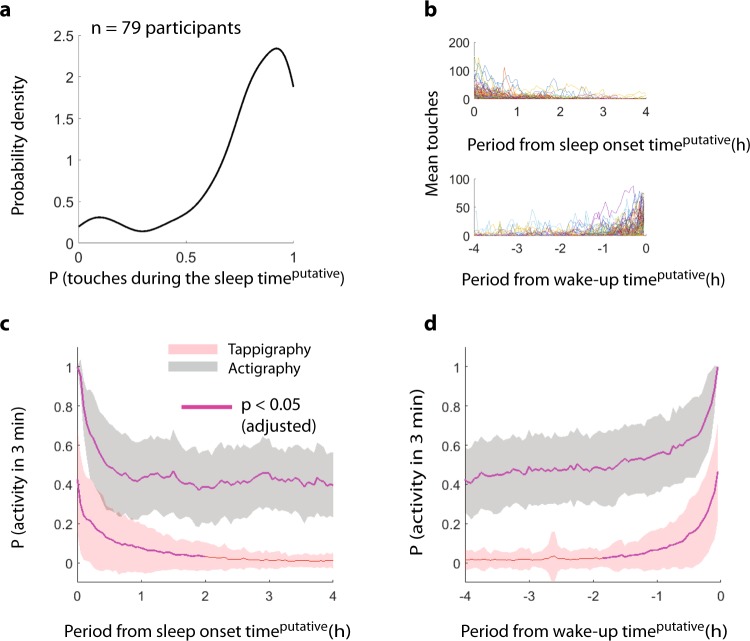
Prevalence of smartphone touches during actigraphy-inferred sleep. **a** Kernel density plot of the probability of observing smartphone touches during ‘sleep’ in the sampled population—based on the percentage of nights with smartphone touches extracted from each participant. **b** The break down of the number of touches generated after sleep onset and before wake-up times with each participant represented using a different line. The probability of detecting smartphone touches and gross body movements in 3 -min bins after sleep onset (**c**) and before wake-up time (**d**). The dark line represents the central tendency of the population, and the shaded area represents the standard deviation. Note, according to the central tendencies, smartphone touches are observed with a probability of ~0.4 right after sleep onset and right before wake-up time. The statistical testing against a probability of 0 was corrected for multiple comparison correction by using Bonferroni correction

**Fig. 5:**
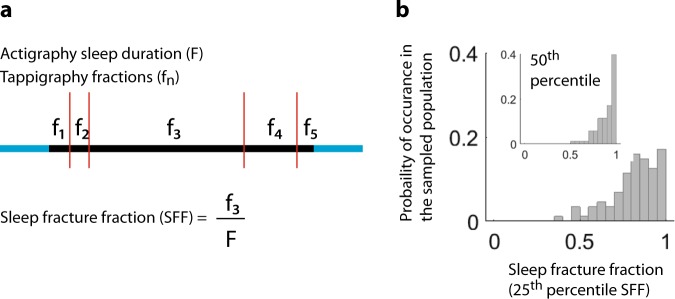
Integrating touchscreen interactions with actigraphy-derived sleep times to quantify sleep disturbances. **a** The metric of sleep fracture fraction (SFF) was derived from each night, which is essentially the longest period of smartphone un-interrupted sleep normalised to the total sleep duration according to actigraphy. **b** The population distribution of the 25th percentile (50th percentile as insert) values of the SFF. Note, SFF of 1 would indicate un-interrupted sleep and SFF of 0.5 indicates continuous sleep accounting for only half of the sleep duration

Some of the observations described above were consistent with the idea that actigraphy can overestimate sleep. If this is the case, smartphone touches must be visible even during the putative sleep times determined by actigraphy. First, we quantified the probability of observing a smartphone touch during the actigraphy-derived sleep. Users were found to be regularly interacting on the phone in the putative sleep period (Fig. 4). Next, we addressed the temporal distribution of the probability of observing smartphone touches in 3 -min bins after the sleep onset and before the wake-up time. This analysis was necessary given that simply comparing the population central tendencies suggested that actigraphy overestimated sleep by 0.4 h. However, neither does that simple comparison indicate which parts of the night are most vulnerable to the phone nor does it capture the low probability disruptions that may consistently occur across the population. According to the temporal distribution analysis, the probability of observing a touch remained significantly greater than 0 for ≈2 h after sleep onset and before wake-up time. Unsurprisingly, the body movements were observed through the night, albeit lower than at sleep onset or wake-up time. The smartphone touchscreen events that interrupted the actigraphy-based sleep could be used to quantify sleep fragmentation or the extent of un-interrupted sleep experienced by the users (Fig. 5). According to this analysis, only ~20% of the sampled population regularly (at 25th percentile, ~40% at 50th percentile) experienced smartphone-free sleep. Scatter plots with probability density estimates were generated using Nils Haentjens’s *scatter_kde* function implemented by using MATLAB.

## Discussion

This study revealed the state of physical activity during the cognitively engaging fine movements captured on the smartphone touchscreen. The smartphone touches consistently occupied the periods of low physical activity, and this has substantial repercussions for sleep. On the one hand it offers a new opportunity to quantify sleep, and on the other hand it offers a highly quantitative insight into the extent of integration of modern digital behaviour with sleep behaviour, in particular with falling asleep and waking up. According to our results, smartphone touches recorded in the background can yield a reliable proxy measure of sleep and, surprisingly, the digital interactions are as much a part of falling asleep as they are a part of waking up.

Firmly understanding the link between smartphone usage and physical activity is a key step for sleep research and medical conditions associated with physical inactivity.^11^ For the former, there is no consensus on to what extent smartphone usage or digital media usage influences sleep, but the conventional observations have vigorously employed self-reports which can be expected to provide a noisier, more time consuming and expensive understanding compared with the sensors used here.^12,13^ We consistently find that smartphone interactions occurred at rest. In this study, we focused on a measure of physical activity that is typically used in resolving the sleep–wake state in the popular Cole–Kripke algorithm.^3^ This measure uses the accelerations recorded at the wrist as raw inputs. A well-known limitation of actigraphy is that while low acceleration values with the watch firmly attached on to the wrist is a reliable indicator of physical rest, the higher values may be contaminated for instance by a bumpy ride in a vehicle. Therefore, when considering our finding that there can be a second peak of smartphone usage with high actigraphy values, we must take the technical limitation of actigraphy into consideration.

We deployed a simplistic algorithm to determine the putative sleep onset and wake-up times based on the smartphone touches. This tappigraphy algorithm essentially combined two safe assumptions. First, the smartphone screen can be only touched when awake. Second, users follow a 24-h sleep–wake cycle. The first assumption provided us with a list of smartphone usage gaps of which at least one contained sleep duration. The second assumption helped select the maximum gap which overlapped with the inactive phase—and this gap was identified as putative sleep. This simple approach resulted in sleep-onset times and wake-up times, which were highly correlated with the times extracted from the standard actigraphy or sleep diary. Admittedly, there is a scope for improvement—neither can the current analysis detect day-time naps nor can we be sure it would accurately reflect sleep in subjects who cannot follow the 24 -h cycle as in shift workers or in sleeping disorders such as insomnia. Day-time naps in particular cannot be reliably captured using actigraphy alone, and the development & validation of nap-specific algorithms based on Tappigraphy must rely on additional behavioural or physiological readouts.^14^ However, our initial version can be a powerful tool for quantifying sleep in individuals who follow diurnal behavioural patterns and addressing its utility in shift workers or sleeping disorders is a necessary next step.

Interestingly, the sleep durations were typically shorter when measured by using tappigraphy in comparison with actigraphy or sleep diaries. The overestimation of sleep by actigraphy (in the order of 15 min) is a well-recognised methodological issue and is typically explained by the delay between reduced motility and falling asleep.^3,10,15^ Our findings suggest a more complex scenario in the sense that there is a highly active period—in terms of cognitively engaging smartphone behaviour—between the two sleep-related landmarks. A surprising finding was the prevalence of smartphone interactions surrounding the wake-up times, suggesting another source of sleep-overestimation in actigraphy. In sum, smartphones occupy the apparently quiescent periods between going to bed and falling asleep, and waking up and getting out of bed. These findings raise the crucial question of whether these periods were used differently in terms of cognitive activity prior to the introduction of smartphones in human behaviour. Regardless, due to the general consensus that a quiescent period before sleep is integral to initiating sleep, combining tappigraphy and actigraphy (or polysomnography) may offer highly relevant measures of sleep hygiene.^16^ Indeed, according to our estimates based on the integrated measure of sleep fracture fraction—that quantifies the extent of actigraphy sleep involving smartphone touchscreen disturbances—only 40% of the users regularly experience undisturbed sleep.

We opportunistically used the demographic data assimilated on age, gender, height and weight to address how they related to sleep measures derived from tappigraphy and actigraphy. In terms of sleep duration, actigraphy-based values did not relate to the demographic information. However, according to tappigraphy, there was a weak link (tendency) to body weight and a strong link to overall phone usage such that both variables negatively correlated with sleep duration. An interesting pattern of the results emerged when we focused on the night-to-night variations in sleep using tappigraphy or actigraphy. Actigraphy revealed that individuals with higher weight showed more irregularity in sleep. In tappigraphy, we found a striking gender difference, with females being more regular sleepers than males. The findings from such a small sample are by no means conclusive, but it does offer an interesting starting point in using these methods to address inter-individual differences and offers a preliminary suggestion that tappigraphy may be sensitive to distinct features compared with actigraphy.

This study offers tappigraphy as a clear complement and reflection to actigraphy in sleep measurements. However, for tappigraphy to replace actigraphy in sleep measures it must be tested against polysomnography. For instance, the pending measures may clarify the physiological states surrounding the near-bedtime and near-rise time touchscreen touches. Interestingly, it would also help compliment the sleep fracture fraction measure derived here: which sleep stages are most susceptible to such fractures? However, as polysomnography requires the laboratory setting tappigraphy may less faithfully reflect day-to-day sleep in such artificial conditions. Another factor pertaining to the application of tappigraphy in sleep measurements is that the current study sample consisted almost exclusively of young adults and a systematic exploration is needed to address if the findings here can be generalised to the rest of the population. Finally, although focusing on the cognitively engaging touchscreen interactions negates false positives when quantifying sleep disturbances, they also make the measurement insensitive to wakeful periods where the person is engaged in activities like watching long videos on the phone or in long phone-free activities, like watching TV while falling asleep. A cross-platform digital logging in addition to tappigraphy may help address this in a next step.

According to this study, smartphones appear truly integrated into modern human behaviour, including into our sleeping habits. Quantifying the extent of integration may not only yield a better understanding of behaviour in the real world but also yield new measures of sleep. The sleep measures introduced here do not rely on high smartphone usage as such, but rather rely on the phenomenon that smartphones are used at rest and that they can be easily used even when in bed. The favourable consequence of this deep digital integration is that we can now develop highly scalable measures of sleep. Whether the integration is detrimental to sleep itself needs to be clarified.

## Methods

### Participants and recruitment

Participants from the campus of Leiden University were recruited by using advertisements on a closed online platform and department-wide emails. Candidates with known neurological and psychiatric diagnosis based on self-reports were excluded from recruitment. Due to technical limitations, those users with an Android operating smartphone were invited to participate and under the condition that the phone remains strictly un-shared during the study period. A total of 88 right-handed participants were recruited (44 females, 16–45 years of age, mean age 23). The experimental procedures used here were approved by the Ethical Committee at the Institute of Psychology at Leiden University. All the participants provided written and informed consent and were compensated for their time using a cash reward or course credits. The weight with one layer of clothing, height and the year/month of birth was collected from each participant. As a part of a larger study, the volunteers also consecutively participated in a range of laboratory assessments beyond the scope of this report.

### Actigraphy measurement

The gross movements (three-axis accelerometer), the ambient light and near-body temperature were measured using GENEACTIV watches (Activinsights, Cambridgeshire, UK). Participants were instructed to wear the watch on both wrists for a minimum of 2 weeks, and the data from the left wrist are primarily presented here. Four participants were unable to follow the instruction due to reported discomfort and were eliminated from the study. Participants who only intermittently removed the watches during part of the observation period were not eliminated, as these periods could be accounted for using the near-body temperature measurements. The watches were set to acquire the data at 50 Hz and the data were recovered after 14 days of acquisition or earlier, only to be reset for continued use if the subjects were willing to participate for an additional week.

### Tappigraphy measurement and on-phone sleep diary

The touchscreen interactions were quantified using the TapCounter App (QuantActions Ltd. Lausanne, Switzerland).^17^ The App was installed by each user from the Google Playstore (Google, Mountain View, USA). The App is designed to gather the precise timestamps of all touchscreen interactions and operates in the background. Only those touchscreen interactions which occurred during the ‘unlocked’ state of the screen were considered here. Each user was provided with a unique user code—and when entered into the App, the data were streamed to the cloud along with the unique code for further processing. All data were encrypted during transmissions. Users were instructed to note the bed, sleep, wake-up and out-of-bed times every day during the actigraphy measurements on a ‘notes’ feature built into the TapCounter (sleep and wake-up reports are were here). The nights after which the participants failed to report these times were eliminated from the analysis pertaining to sleep diaries versus tappigraphy, and sleep diaries versus actigraphy. The app failed to operate in five participants due to missing device permissions.

### Participant instructions

Participants were not explicitly made aware that any analysis linking actigraphy, sleep diaries and tappigraphy prior to the de-briefing at the end of the observation period. Through the observation period, the participants were under the impression that the sleep-related variables from the watches and diaries would be linked to the laboratory measures obtained towards a broad study on sensorimotor properties. Towards actigraphy, the participants were instructed to wear the watches throughout the observation period (24 h/day). No instruction was given as to where to place the smartphone (as in next to the bed or not) in the same period. Towards the sleep diaries, the participants were instructed to use the ‘notes’ feature on the TapCounter App whenever convenient, but in the morning period to report on the previous night’s sleep using a single entry per day. The timestamps of these notes were further recorded to screen for any consistent patterns in the note-taking behaviour or to address whether participants adhered to the instructions.

### Actigraphy algorithm

The accelerations gathered along the three axes by the actigraphy watches were combined using the sum of squares and low-pass filtered at 2 Hz. To estimate the putative sleep and wake times, we employed the standard Cole–Kripke algorithm on the filtered data with slight modifications.^3^ A key part of this algorithm—the minute-by-minute categorisation of the data into rest-active states based on the weighted sum of the current minute with that of the surrounding minutes—was extracted to study the physical activity state during smartphone usage. The algorithm was implemented on MATLAB (MathWorks, Natick, USA) and used pre-existing codes.^18^ We modified the codes such that the automatic scoring of sleep and wake by the Cole–Kripke algorithm was further checked by the near-body temperature and ambient light measurements. Firstly, any putative sleep period where the median temperature dropped below 25 °C was ignored— removing instances where the user removed the watch from the body. Secondly, any putative sleep period where the median ambient light levels failed to drop below 25 lux was ignored and thus restricting the analysis to nighttime sleep and ignoring day-time naps. Thirdly, the putative sleep times had to contain a 10% (36 min) overlap with the 6 -h low-activity period determined using a 24 -h sinewave fit (Casey Cox’s *cosinor* function implemented in MATLAB).^19^ This final step is commonly substituted using sleep diaries, but our approach avoided mixing the subjective diary entries with the objective measurements to determine sleep durations—ensuring the estimated durations are entirely objective.

### Tappigraphy algorithm

The raw touchscreen timestamps were parsed into MATLAB using the parser extractTaps (QuantActions Ltd. Lausanne, Switzerland). The touchscreen timestamps were then processed using a separate algorithm (*getresttimesphone*, implemented in MALTAB) designed to gather the gaps in smartphone use at the circadian rest phase (i.e., at the putative night). To elaborate on this algorithm, first, the phone data were reduced to binary states in 60-s bins (1 as active and 0 as rest). The activity was further processed using a cut-off (5% in an hour threshold) such that the brief periods of activity surrounded by inactivity were labelled as rest. Next, we extracted all of the continuous gaps in smartphone activity, such that the gap in usage was greater than an arbitrarily set 2 h threshold. In a parallel set of computations, we obtained a 24 -h sinewave fit on the time series of smartphone interactions using the Cosinor analysis (Casey Cox’s *cosinor* function implemented in MATLAB).^19^ This sinewave fit was then used to determine the 6-h long periods with the least activity in the tapping data in 24 -h windows. The two parallel streams were combined to select those activity gaps which had a minimum of arbitrarily set 10% overlap (36 min) with the 6 h period, and these gaps were labelled as ‘sleep’.

### Statistical analysis

Simple linear regressions (using the bi-square fitting method, implemented using the *fitlm* function in MATLAB) were employed for all of the analysis, except that for the analysis including demographic information where multiple regression was used. The simple pairwise regression was the method of choice where the relationship between a traditional parameter—from actigraphy or sleep diary—was compared against the output of tappigraphy.^20^ These paired comparisons were conducted on concatenated data with the resolution of each night and with non-concatenated data with the resolution of each individual. The concatenation for bed times, wake-up times and sleep duration, from one subject say *S*_*1*_ with another *S*_*2*_ say for an estimate *e*: *S*_*c*_ = *S*_1_ *e*_*i*∈{1,...,*m*}_ ∥*S*_2_ *e*_*i*∈{1,...,*n*}_, where *m* and *n* are the number of nights recorded for *S*_*1*_ and *S*_*2*_, respectively, and *S*_*c*_ is the concatenated data. The subsequent subject’s data, say *S*_*3*_ was concatenated to *S*_*c*_ and so on. For bed times and wake-up times, to enable the correlations of values from a 24 h clock in a linear space a simple transformation for the sleep onset values under 10 am, such that 01 h in past midnight was considered as 25 h. Non-concatenated data were used to study inter-individual differences and given the importance of central tendency estimate of sleep (median sleep duration, and the corresponding coefficient of variation, CoV), we used tappigraphy versus actigraphy paired comparisons.^21^ Pairwise regression was also used to address the dependency of measurement error in tappigraphy (evaluated against actigraphy) versus smartphone usage. The simple regression method was also used to address how much phone use is needed to obtain useful tappigraphy-based estimates (therefore serving to establish an inclusion criterion in future studies based on phone usage alone).

Multiple regression was used in the explorative analysis of how the demographic information was related to the key sleep metrics of sleep median and CoV. The purpose of this analysis was to illustrate the nature of the relationships that may be discovered when using tappigraphy or actigraphy independently. Four different regression models were tested with the following dependent variables: actigraphy-based median sleep duration and CoV, and tappigraphy-based median sleep duration and CoV. Towards all of these models, the following explanatory variables were used: phone usage (measured as the number of touches per day), age, gender (dummy variable), height and weight. Each model was used to test five hypotheses simultaneously, and the corresponding *t* test *α* (set at 0.05) was Bonferroni corrected for this multiple comparison (the values which were >α post correction, but <0.05 are still noted in the results and indicated as such due to the exploratory nature of this analysis). In these multiple regressions, the two subjects with ages higher than 35 were excluded as outliers (>5 STD from the mean age). The subject elimination is detailed in the Supplementary Table, and the statistics in the results section is reported with the corresponding degrees of freedom. When MATLAB estimated *p*-value was at 0, then *p* < 0.0001 is used to describe the results.

Paired *t* test was used to compare the median sleep durations (from each individual) obtained using actigraphy versus tappigraphy in the sampled population (*α* = 0.05). In a separate analysis, one sample *t* tests of mean probability of phone interactions in 3 -min bin versus 0 were conducted to establish the dynamics of interrupted nights (*α* = 0.05, Bonferroni corrected).

### Reporting summary

Further information on experimental design is available in the Nature Research Reporting Summary linked to this article.

## Supplementary information

Supplementary Table and Figures

Reporting Summary

## Data Availability

The processed data sets used towards this report are available at https://dataverse.nl.

## Supplementary information

**Supplementary Information** accompanies the paper on the *npj Digital Medicine* website (10.1038/s41746-019-0147-4).

## Code availablilty

The main codes developed and used in this report are available at https://dataverse.nl. The codes were developed and operate on MATLAB 2017b (Mathworks, Natick, USA).
