# Supplementary material for: Capturing sleep–wake cycles by using day-to-day smartphone touchscreen interactions
Source: NPJ Digit Med. 2019 Jul 29;2:73. doi: 10.1038/s41746-019-0147-4 (PMC6662846; doi:10.1038/s41746-019-0147-4)
Supplement: Supplementary file 1 — Supplementary Table and Figures [file 41746_2019_147_MOESM1_ESM.pdf]

Reason for elimination	Subjects eliminated	Analysis impacted	Subjects remaining in analysis from N = 88
Dysfunctional App	5	All	79
Removed watches	4	All	
No values activity values at Cole-Kripke D > 7	5	Linear regression linking D values at the extreme	74
Age-related outliers	2	Multiple linear regression on inter-individual differences	77

Supplementary Table: A summary of the subjects eliminated post data acquisition.

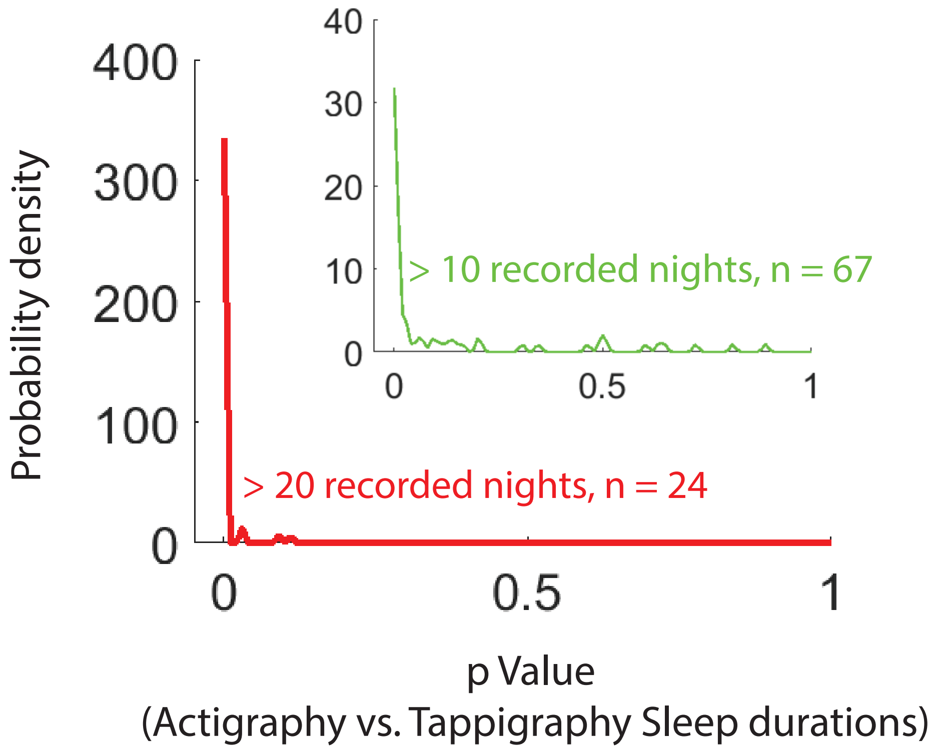

Supplementary Figure 1: The population distribution of the robust-linear regression estimated p values, with the regression performed from the data generated by each participant. The KS-probability density of the sampled population is plotted at two different thresholds (> 10 nights and > 20 nights).

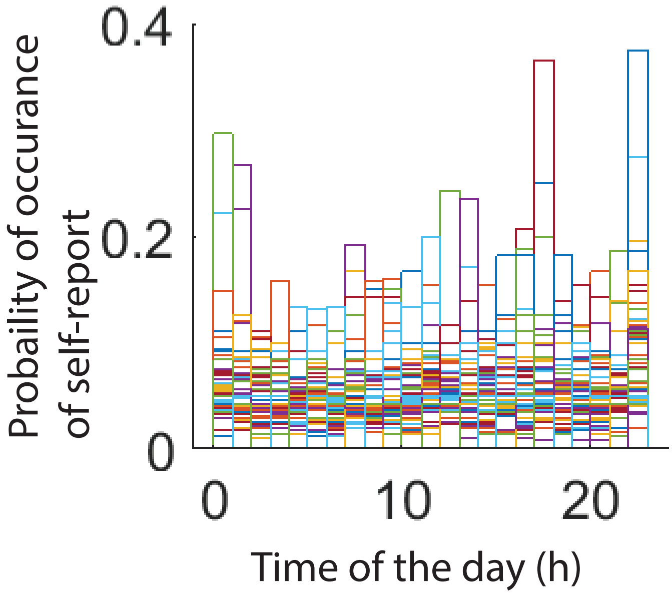

Supplementary Figure 2: The overlayed histograms of the sleep-diary reporting times, with each individual, plotted with a distinct line and color.
